# 청구ㆍ사망자료 기반 심뇌혈관질환 국가통계 생산체계: 발생 규모와 치명률, 2011–2023년 - 이차 출판

**DOI:** 10.56786/PHWR.2026.19.40.2_K

**Published:** 2026-09-21

**Authors:** 서정 신, 재원 길, 소정 신, 재용 이, 민성 조, 나연 안, 상경 이, 영창 조, 승찬 유, 연희 유, 승준 이, 장환 배, 민 김, 규선 염, 장현 백, 호규 이, 현창 김

**Affiliations:** 1 연세대학교 디지털헬스연구원; 2 고려대학교 의과대학 의생명정보학교실; 3 연세대학교 일반대학원 보건학과; 4 연세대학교 의과대학 예방의학교실; 5 연세대학교 의과대학 의생명시스템정보학교실; 6 질병관리청 만성질환관리국 만성질환관리과; 7 연세대학교 의과대학 세브란스 심장혈관병원 심장내과; 8 좋은삼선병원 내과; 9 성균관대학교 의과대학 강북삼성병원 내과; 10 충북대학교 의과대학 신경과학교실; 11 성균관대학교 의과대학 신경과학교실; 12 연세대학교 의과대학 내과학교실

**Keywords:** 급성심근경색증, 뇌졸중, 발생률, 치명률, 감시체계

## Abstract

**목적:**

급성심근경색증과 뇌졸중은 우리나라의 주요 사망 및 질병부담 원인으로 국가 차원의 지속적인 감시가 필요한 질환이다. 본 보고는 심뇌혈관질환 국가통계 생산체계의 운영 개요를 소개하고, 이를 통해 산출한 2011–2023년 급성심근경색증 및 뇌졸중의 발생 규모와 치명률 현황을 제시하고자 하였다.

**방법:**

국민건강보험공단 자료에서 2002–2023년 급성심근경색증 및 뇌졸중 관련 입원 사례를 추출하였다. 사망 여부와 사망일은 국민건강보험공단 자격자료에서, 사망원인은 국가데이터처 사망원인자료와의 연계를 통해 확인하였다. 2002–2010년은 washout 기간으로 설정하고, 2011–2023년 발생 사건을 대상으로 통계를 산출하였다. 사건은 진단코드, 입원정보, 검사, 영상검사, 시술 및 사망정보를 포함한 검증된 알고리즘으로 식별하였고, 알고리즘의 타당도는 주기적인 의무기록조사를 통해 평가하였다. 발생 건수와 발생률은 최초 발생, 재발, 전체 발생으로 구분하여 산출하였다. 치명률은 입원 후 30일 및 1년 내 모든 원인 사망을 기준으로 산출하고 연령군별로 제시하였다.

**결과:**

2011–2023년 급성심근경색증 총 발생 건수는 22,395건에서 34,768건으로, 뇌졸중 총 발생 건수는 99,837건에서 113,098건으로 증가하였다. 재발 건수도 급성심근경색증은 1,472건에서 3,373건으로, 뇌졸중은 16,925건에서 22,813건으로 증가하였다. 연령군별 30일 및 1년 치명률은 두 질환 모두 80세 이상에서 가장 높았다. 2023년 의무기록조사 결과, 사건 식별 알고리즘의 양성예측도는 최초 급성심근경색증 92.2%, 재발 급성심근경색증 78.2%, 최초 뇌졸중 88.1%, 재발 뇌졸중 81.0%였다.

**결론:**

국민건강보험공단 청구ㆍ자격자료와 국가데이터처 사망원인자료를 활용한 심뇌혈관질환 국가통계 생산체계를 통해 급성심근경색증과 뇌졸중의 발생 규모와 치명률을 지속적으로 산출하였으며, 주기적인 의무기록조사를 통해 통계 생산 방법의 타당성을 확인하였다. 본 생산체계는 국가 단위 심뇌혈관질환 질병부담을 지속적으로 감시하고 근거 기반 예방ㆍ관리 정책을 지원하는 기반으로 활용될 수 있다.

**핵심요약**

**① 이전에 알려진 내용은?**

사망통계만으로는 비치명적 사건과 재발을 포함한 급성심근경색증 및 뇌졸중의 전체 발생 규모를 파악하는 데 한계가 있다.

**② 새로이 알게 된 내용은?**

국민건강보험공단 청구ㆍ자격자료와 국가데이터처 사망원인자료를 활용하고, 주기적인 의무기록조사를 통해 사건 식별 알고리즘의 타당도를 점검하는 국가통계 생산체계를 운영하고 있다. 이를 통해 최초 발생과 재발을 포함한 발생 규모와 치명률을 지속적으로 산출하였다.

**③ 시사점은?**

행정자료와 의무기록조사를 결합한 통계 생산체계는 국가 단위 심뇌혈관질환 발생 규모와 치명률을 효율적으로 모니터링하는 기반으로 활용될 수 있다.

## 서 론

급성심근경색증과 뇌졸중은 우리나라의 대표적인 심뇌혈관질환으로, 질병부담 변화를 지속적으로 파악하고 예방ㆍ관리 정책의 근거를 마련하기 위해 국가 단위 감시가 필요한 질환이다[1]. 기존 사망통계는 질환으로 인한 사망 규모를 파악하는 데 유용하지만, 비치명적 사건과 재발 사건을 포함한 전체 발생 규모를 평가하는 데에는 한계가 있다. 따라서 급성심근경색증과 뇌졸중의 발생 규모와 치명률을 표준화된 방식으로, 지속적으로 산출할 수 있는 국가통계 생산체계가 필요하다.

우리나라는 전 국민 건강보험 체계를 기반으로 전 국민 수준의 의료이용 자료를 구축하고 있어, 국민건강보험공단 청구자료와 사망자료를 연계하면 급성심근경색증과 뇌졸중의 발생 규모와 발생 후 치명률을 효율적으로 모니터링할 수 있다. 다만 청구자료는 진료비 청구를 목적으로 수집된 자료이므로, 진단코드만으로는 실제 신규 발생 사건과 기존 질환의 지속적 관리 또는 재입원을 구분하기 어렵다. 이에 심뇌혈관질환 국가통계 생산체계에서는 표준화된 사건 식별 알고리즘을 적용하고, 주기적인 의무기록조사를 통해 알고리즘의 타당도를 확인하는 절차를 병행하고 있다[2,3].

본 보고는 질병관리청의 「심뇌혈관질환 국가통계 생산체계」 운영 사업의 개요를 소개하고, 국민건강보험공단 청구ㆍ자격자료와 국가데이터처 사망원인자료를 활용하여 산출한 2011–2023년 급성심근경색증 및 뇌졸중의 발생 규모와 치명률 현황을 제시하고자 한다.

## 방 법

### 1. 자료원

자료원으로는 국민건강보험공단의 전 국민 건강정보 데이터베이스를 활용하였다. 이 자료는 인구학적 특성, 건강보험 자격정보, 의료이용 정보, 진단 및 처치 내역 등을 포함한다. 2002년 1월 1일부터 2023년 12월 31일까지 급성심근경색증 또는 뇌졸중 관련 진단코드가 부여된 입원자료를 추출하였다. 사망 여부와 사망일은 국민건강보험공단 자격자료를 통해 2024년 12월 31일까지 확인하였고, 사망원인은 국가데이터처 사망원인자료와의 연계를 통해 확인하였다.

신규 발생 사례를 정의하기 위해 2002–2010년을 washout 기간으로 설정하였으며, 최종 분석은 2011–2023년 발생 사건을 대상으로 하였다. 본 연구는 익명화된 2차 자료를 활용하였으며, 연세대학교 의과대학 연구윤리심의위원회 승인을 받았다.

### 2. 급성심근경색증 및 뇌졸중 사건 식별

하나의 질병 사건에 대해 장기 입원, 전원, 반복 청구 등이 발생할 수 있으므로, 관련 진단코드가 퇴원진단으로 기록된 연속 청구 건을 통합하여 입원 에피소드를 구성하였다. 이후 동일 환자에게 추가 입원이 발생한 경우, 두 입원의 최초 입원일 간 간격이 28일을 초과하고 이전 입원 퇴원일과 다음 입원 입원일 사이 간격이 3일 이상인 경우 새로운 에피소드로 간주하였다.

급성심근경색증은 International Classification of Diseases, Tenth Revision (ICD-10) 코드 I21–I23, 즉 급성심근경색증(I21), 후속심근경색증(I22), 급성심근경색증 후 특정 현존 합병증(I23)을 기준으로 하였으며, 진단 정확도를 높이기 위해 심전도, 심근효소 검사, 관상동맥조영술, 경피적관상동맥중재술, 관상동맥우회술 등 급성 심장질환 평가 또는 치료 관련 정보, 입원기간 및 사망정보를 함께 활용하였다. 뇌졸중은 ICD-10 코드 I60–I61, I63–I64, 즉 지주막하출혈(I60), 뇌내출혈(I61), 뇌경색증(I63), 출혈 또는 경색증으로 명시되지 않은 뇌졸중(I64)을 기준으로 하였으며, computed tomography, magnetic resonance imaging, magnetic resonance angiography 등 뇌영상검사와 관련 치료 및 시술 정보, 입원기간 및 사망정보를 함께 고려하였다.

사례 정의를 만족하는 첫 번째 입원 에피소드를 최초 발생 사건으로 정의하였고, 이후 동일 환자에서 다시 사례 정의를 만족하는 에피소드는 재발 사건으로 분류하였다. 청구자료 기반 사건 식별 알고리즘의 타당도는 표본 의무기록조사를 통해 평가하였다. 의무기록조사는 알고리즘으로 식별된 급성심근경색증 및 뇌졸중 의심 사건을 대상으로 하였으며, 조사자가 해당 입원 에피소드의 의무기록을 검토하여 임상ㆍ역학적 기준에 따른 실제 사건 여부를 판정하였다.

의무기록조사 대상 의료기관은 의료기관별 급성심근경색증 및 뇌졸중 진료 실적 등을 기준으로 후보 기관을 선정한 후 조사 참여를 요청하였으며, 참여 의사를 밝힌 기관 중 전국 권역과 의료기관 종별 분포를 고려하여 최종 선정하였다. 최종 조사에는 총 24개 의료기관(상급종합병원 5개, 종합병원 11개, 병원 8개)이 참여하였다. 급성심근경색증 1,399건과 뇌졸중 1,741건이 포함되었으며, 알고리즘으로 식별된 사건 중 의무기록 검토를 통해 실제 사건으로 판정된 비율을 양성예측도로 산출하였다. 또한 매년 1,000건 이상의 사례에 대해 의무기록을 검토하여 사건 식별 알고리즘의 타당도를 지속적으로 점검하고 있다. 동일한 국가통계 생산체계의 초기 적용 결과로 2011–2020년 뇌졸중 및 급성심근경색증 발생과 치명률이 보고된 바 있다[4,5].

### 3. 통계 분석

2011–2023년 동안 급성심근경색증과 뇌졸중의 발생 건수, 발생률, 치명률을 산출하였다. 발생 건수와 발생률은 최초 발생, 재발, 전체 발생으로 구분하여 산출하였으며, 전체 발생은 최초 발생과 재발의 합으로 정의하였다. 발생률은 인구 10만 명당 조발생률과 연령표준화 발생률로 제시하였다. 연령표준화는 2005년 한국 표준인구를 이용한 직접표준화법을 적용하였다. 치명률은 국민건강보험공단 자격자료에서 확인한 사망 여부와 사망일을 이용하여 입원 후 30일 및 1년 내 모든 원인 사망을 기준으로 산출하였다. 연령군별 치명률은 40–64세, 65–79세, 80세 이상으로 구분하여 제시하였다.

## 결 과

### 1. 국가통계 생산체계

「심뇌혈관질환 국가통계 생산체계」는 급성심근경색증과 뇌졸중의 발생 규모, 발생률 및 치명률을 표준화된 방식으로 산출하기 위한 질병관리청 사업이다. 통계 산출을 위해 먼저 국민건강보험공단 청구ㆍ자격자료를 확보하여 급성심근경색증 및 뇌졸중 관련 입원 에피소드를 구성하고, 사건 식별 알고리즘을 적용하여 최초 발생 및 재발 사건을 식별하였다. 이후 국민건강보험공단 자격자료에서 사망 여부와 사망일을 확인하고 국가데이터처 사망원인자료를 활용하였으며, 표본 의무기록조사를 통해 사건 식별 알고리즘의 타당도를 평가하였다. 이러한 과정을 거쳐 발생 건수, 발생률 및 치명률을 산출하고 연도별 결과를 검토하였다. 이 생산체계를 통해 2011–2023년 급성심근경색증 및 뇌졸중의 발생 건수, 발생률 및 치명률을 산출하였다.

### 2. 사건 식별 알고리즘의 양성예측도

2023년 의무기록조사 결과, 사건 식별 알고리즘의 양성예측도는 최초 급성심근경색증 92.2%, 재발 급성심근경색증 78.2%, 최초 뇌졸중 88.1%, 재발 뇌졸중 81.0%였다(표 1) [3]. 이는 본 보고에서 사용한 청구자료 기반 알고리즘이 국가 단위 급성심근경색증 및 뇌졸중 발생 현황을 모니터링하는 데 활용 가능한 수준의 타당도가 있음을 보여준다.

### 3. 급성심근경색증 및 뇌졸중 발생 규모

2011–2023년 동안 급성심근경색증 발생 건수는 일부 연도별 변동에도 불구하고 전반적으로 증가하였다(그림 1). 총 발생 건수는 2011년 22,395건에서 2023년 34,768건으로 증가하였고, 최초 발생 건수는 20,923건에서 31,395건으로 증가하였다. 재발 건수는 1,472건에서 3,373건으로 증가하여, 전체 급성심근경색증 사건 중 재발 사건의 비율은 2011년 6.6%에서 2023년 9.7%로 증가하였다. 성별로는 전 기간 동안 남성의 발생 건수가 여성보다 많았으며, 남녀 발생 건수 비는 2011년 2.14에서 2023년 2.96으로 증가하였다.

뇌졸중 발생 건수도 일부 연도별 변동에도 불구하고 2011년보다 2023년에 많았다(그림 1). 총 발생 건수는 2011년 99,837건에서 2023년 113,098건으로 증가하였고, 최초 발생 건수는 82,912건에서 90,285건으로 증가하였다. 재발 건수는 16,925건에서 22,813건으로 증가하였으며, 2011년과 2023년을 비교했을 때 재발 건수의 증가율은 최초 발생 건수보다 컸다. 성별로는 뇌졸중도 남성의 발생 건수가 여성보다 많았으나, 급성심근경색증에 비해서는 남녀 차이가 작았다.

연령별 발생 규모를 살펴보면, 급성심근경색증은 2023년 60–69세 연령군에서 9,605건으로 가장 많은 발생 건수를 보였고, 80세 이상 연령군의 발생 건수는 2011년 3,472건에서 2023년 7,276건으로 증가하였다. 뇌졸중은 2011년 70–79세 연령군에서 발생 건수가 31,168건으로 가장 많았으나, 2023년에는 80세 이상 연령군이 34,635건으로 가장 많은 발생 건수를 보였다.

### 4. 급성심근경색증 및 뇌졸중의 치명률

전체 연령군에서 산출한 최초 발생 기준 30일 치명률은 급성심근경색증의 경우 2011년 9.8%에서 2023년 9.1%로 감소하였고, 뇌졸중의 경우 2011년 9.0%에서 2023년 7.8%로 감소하였다. 최초 발생 기준 1년 치명률은 급성심근경색증에서 2011년 17.1%에서 2023년 16.0%로 감소하였고, 뇌졸중에서 2011년 20.3%에서 2023년 19.3%로 감소하였다.

연령군별로는 급성심근경색증과 뇌졸중 모두 80세 이상 연령군에서 30일 및 1년 치명률이 가장 높았다(그림 2). 급성심근경색증의 30일 및 1년 치명률은 고령층에서 높은 수준을 보였으나, 일부 연도별 변동에도 불구하고 연구 기간 동안 전반적으로 감소하였다. 특히 80세 이상 연령군에서 30일 치명률은 2011년 24.1%에서 2023년 19.9%로, 1년 치명률은 2011년 42.5%에서 2023년 36.2%로 감소하였다.

뇌졸중의 경우에도 80세 이상 연령군에서 치명률이 가장 높았으며, 2019년 이후 증가가 관찰되었다. 특히 80세 이상 연령군의 30일 치명률은 2019년 11.2%에서 2022년 12.5%로 증가한 뒤, 2023년에는 11.9%로 소폭 감소하였으나 2019년보다는 높은 수준이었다. 1년 치명률은 2019년 32.5%에서 2022년 36.2%로 증가한 뒤, 2023년에는 35.0%로 소폭 감소하였다.

## 결 론

본 보고는 국민건강보험공단 청구ㆍ자격자료와 국가데이터처 사망원인자료를 활용한 심뇌혈관질환 국가통계 생산체계의 운영 방식과 산출 결과를 제시하였다. 이 체계를 통해 2011–2023년 급성심근경색증과 뇌졸중의 최초 발생 및 재발을 포함한 발생 규모와 30일 및 1년 치명률을 지속적으로 산출하였다. 해당 기간 두 질환의 절대 발생 건수는 증가하였으며, 치명률은 연령에 따라 차이를 보여 80세 이상에서 가장 높았다. 본 보고는 국가통계 생산체계에서 산출된 지표를 기술한 조사/감시 보고로, 관찰된 변화의 원인을 직접 평가하지는 않았다. 다만 2019년 이후 일부 치명률 지표에서 관찰된 변화는 코로나바이러스감염증-19 유행 시기의 의료이용 변화, 응급진료 접근성, 입원 환자의 중증도 변화 등과 관련되었을 가능성이 있으며, 이에 대한 인과적 해석을 위해서는 추가적인 분석 연구가 필요하다. 이와 함께 주기적인 의무기록조사를 통해 사건 식별 알고리즘의 양성예측도를 확인함으로써 청구자료 기반 통계 생산 방법의 타당도를 점검하고 있다. 본 생산체계는 국가 단위 심뇌혈관질환 발생 규모와 치명률을 효율적이고 지속적으로 모니터링하고, 예방ㆍ관리 정책 수립을 지원하는 기반으로 활용될 수 있다.

## Figures and Tables

**그림 1:**
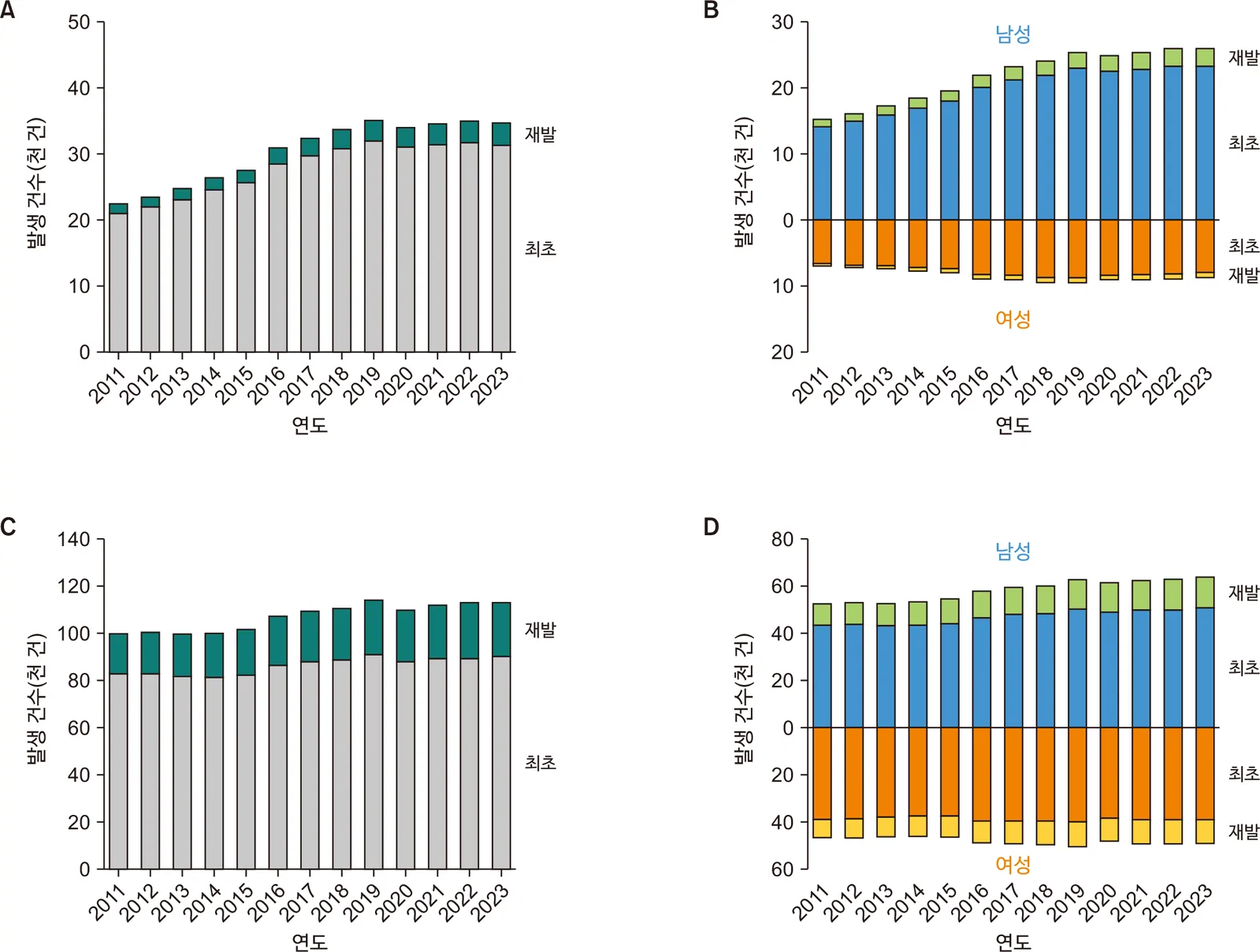
2011–2023년 우리나라 급성심근경색증 및 뇌졸중 발생 건수 추이 (A) 급성심근경색증의 전체 발생 건수(최초 발생 및 재발), (B) 급성심근경색증의 성별 발생 건수(최초 발생 및 재발), (C) 뇌졸중의 전체 발생 건수(최초 발생 및 재발), (D) 뇌졸중의 성별 발생 건수(최초 발생 및 재발). Adapted from the article of Khil et al. (Korean Circ J 2027;57:e40) [2].

**그림 2:**
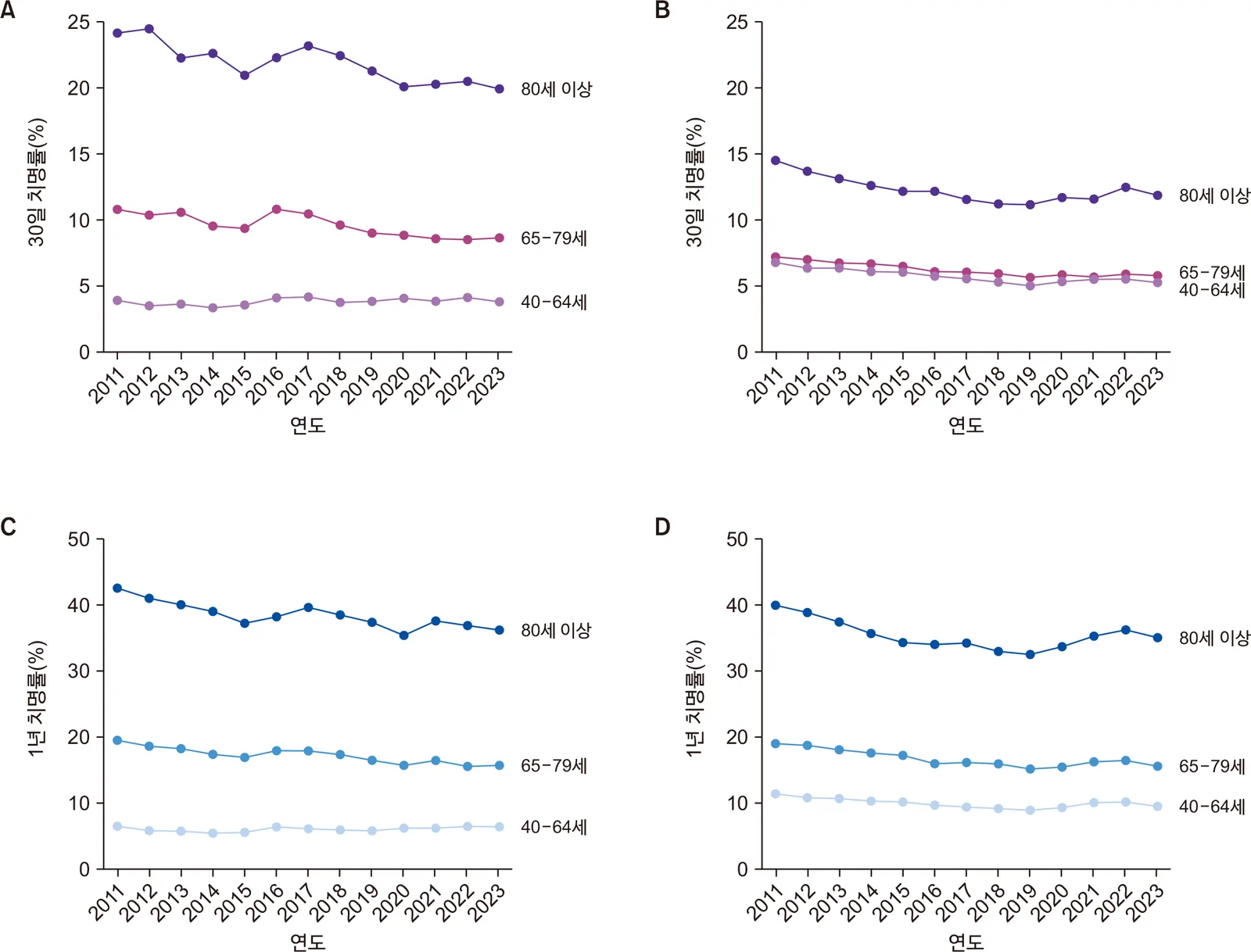
2011–2023년 우리나라 급성심근경색증 및 뇌졸중의 연령군별 치명률 추이 (A) 급성심근경색증의 연령군별 30일 치명률, (B) 뇌졸중의 연령군별 30일 치명률, (C) 급성심근경색증의 연령군별 1년 치명률, (D) 뇌졸중의 연령군별 1년 치명률. Adapted from the article of Khil et al. (Korean Circ J 2027;57:e40) [2].

**표 1:** 2023년 급성심근경색증 및 뇌졸중 사건 식별 알고리즘의 양성예측도

질환	의료기관 종류	질환 유형						
		최초 발생				재발		
		역학적 판정	식별 알고리즘	양성예측도		역학적 판정	식별 알고리즘	양성예측도
급성심근경색증								
	상급종합병원	584	617	94.7%		88	108	81.5%
	종합병원	339	368	92.1%		138	184	75.0%
	1차 의료기관	51	121	42.1%		0	1	0.0%
	가중 통합a)	-	-	92.2%		-	-	78.2%
뇌졸중								
	상급종합병원	458	549	83.4%		137	181	75.7%
	종합병원	335	356	94.1%		154	173	89.0%
	1차 의료기관	275	392	70.2%		57	90	63.3%
	가중 통합a)	-	-	88.1%		-	-	81.0%

-=not available. ^a)^최상위 의료기관 종별을 기준으로 가중 통합함. Adapted from the article of Khil et al. (Korean Circ J 2027;57:e40) [2].
