# Supplementary material for: 청구ㆍ사망자료 기반 심뇌혈관질환 국가통계 생산체계: 발생 규모와 치명률, 2011–2023년 - 이차 출판
Source: Public Health Wkly Rep. 2026 Sep 21;19(40):1701–11. [Article in Korean] doi: 10.56786/PHWR.2026.19.40.2_K (PMC13691640; doi:10.56786/PHWR.2026.19.40.2_K)
Supplement: Translation

# Translation: National Cardiovascular Disease Statistics Production System Based on Claims and Mortality Data: Incidence and Fatality, 2011–2023 - Secondary Publication

**Authors:** Seojeong Shin, Jaewon Khil, Sojung Shin, Jaeyong Lee, Minsung Cho, Na Yeon Ahn, Sang Gyeong Lee, Yeongchang Jo, Seng Chan You, Yeonhee Yoo, Seung-Jun Lee, Jang-Whan Bae, Min Kim, Kyu Sun Yum, Jang-Hyun Baek, Hokyou Lee, Hyeon Chang Kim

**Affiliations:** 1 Yonsei Institute for Digital Health, Yonsei University Seoul Korea; 2 Department of Biomedical Informatics, Korea University College of Medicine Seoul Korea; 3 Department of Public Health, Yonsei University Graduate School Seoul Korea; 4 Department of Preventive Medicine, Yonsei University College of Medicine Seoul Korea; 5 Department of Biomedical Systems Informatics, Yonsei University College of Medicine Seoul Korea; 6 Division of Chronic Disease Control, Department of Chronic Disease Prevention and Control, Korea Disease Control and Prevention Agency Cheongju Korea; 7 Division of Cardiology, Severance Cardiovascular Hospital, Yonsei University College of Medicine Seoul Korea; 8 Department of Internal Medicine, Good Samsun Hospital Busan Korea; 9 Department of Internal Medicine, Kangbuk Samsung Hospital, Sungkyunkwan University School of Medicine Seoul Korea; 10 Department of Neurology, Chungbuk National University College of Medicine Cheongju Korea; 11 Department of Neurology, Sungkyunkwan University School of Medicine Seoul Korea; 12 Department of Internal Medicine, Yonsei University College of Medicine Seoul Korea

**Keywords:** Acute myocardial infarction, Stroke, Incidence, Fatality, Surveillance

## Abstract

**Objectives:**

This report described the National Cardiovascular Disease Statistics Production System and presented the incidence and case fatality rates of acute myocardial infarction (AMI) and stroke in the Republic of Korea from 2011 to 2023.

**Methods:**

Hospitalizations related to AMI and stroke from 2002 to 2023 were identified using data from the National Health Insurance Service (NHIS). Death status and dates of death were determined using NHIS eligibility data, while causes of death were obtained from the Causes of Death Statistics of the Ministry of Data and Statistics. The period from 2002 to 2010 was used as a washout period. Validated algorithms incorporating diagnostic codes, hospitalization duration, tests, imaging, procedures, and mortality information were used to identify first-ever and recurrent events. The validity of the algorithms was assessed through periodic medical record reviews. Crude and age-standardized incidence rates were calculated. Case fatality was defined as all-cause mortality occurring within 30 days and 1 year after hospitalization and was presented by age group.

**Results:**

From 2011 to 2023, the number of AMI events increased from 22,395 to 34,768, while the number of stroke events increased from 99,837 to 113,098. During the same period, recurrent AMI events increased from 1,472 to 3,373 and recurrent stroke events from 16,925 to 22,813. For both diseases, 30-day and 1-year case fatality rates were highest among adults aged 80 years and older. The 2023 medical record review demonstrated positive predictive values of 92.2% for first-ever AMI, 78.2% for recurrent AMI, 88.1% for first-ever stroke, and 81.0% for recurrent stroke.

**Conclusions:**

This system produced national incidence and case fatality statistics for AMI and stroke using NHIS claims and eligibility data, the Causes of Death Statistics, and validated event-identification algorithms. The resulting statistics support ongoing disease surveillance and evidence-based policies for the prevention and management of AMI and stroke.

**Key messages**

**① What is known previously?**

Mortality statistics alone are insufficient to fully capture the overall burden of acute myocardial infarction and stroke, including nonfatal and recurrent events.

**② What new information is presented?**

The national statistics production system uses National Health Insurance Service claims and eligibility data together with the Causes of Death Statistics, validates event-identification algorithms through medical record review, and continuously produces first-ever/recurrent event counts and case fatality rates.

**③ What are implications?**

A statistics production system that combines administrative data with medical record-based validation can provide an efficient platform for monitoring the national burden of cardiovascular diseases and their associated case fatality rates.

## Figures and Tables

**Figure 1:** Number of incident cases of acute myocardial infarction and stroke in the Republic of Korea, 2011–2023 (A) Overall acute myocardial infarction events by first-ever and recurrent events; (B) sex-specific acute myocardial infarction events by first-ever and recurrent events; (C) overall stroke events by first-ever and recurrent events; (D) sex-specific stroke events by first-ever and recurrent events. Recur=recurrent. Adapted from the article of Khil et al. (Korean Circ J 2027;57:e40) [2].

**Figure 2:** Age-stratified case fatality rates of acute myocardial infarction and stroke in the Republic of Korea, 2011–2023 (A) Thirty-day case fatality rate for acute myocardial infarction; (B) thirty-day case fatality rate for stroke; (C) one-year case fatality rate for acute myocardial infarction; (D) one-year case fatality rate for stroke. Adapted from the article of Khil et al. (Korean Circ J 2027;57:e40) [2].

**Table 1:** PPVs of the algorithms for identifying acute myocardial infarction and stroke events, 2023

Disease	Hospital types	Type of disease						
		First				Recurrent		
		Epidemiological determination	Identification algorithm	PPV		Epidemiological determination	Identification algorithm	PPV
Acute myocardial infarction								
	Tertiary hospitals	584	617	94.7%		88	108	81.5%
	Secondary hospitals	339	368	92.1%		138	184	75.0%
	Primary hospitals	51	121	42.1%		0	1	0.0%
	Weighta)	-	-	92.2%		-	-	78.2%
Stroke								
	Tertiary hospitals	458	549	83.4%		137	181	75.7%
	Secondary hospitals	335	356	94.1%		154	173	89.0%
	Primary hospitals	275	392	70.2%		57	90	63.3%
	Weighta)	-	-	88.1%		-	-	81.0%

PPV=positive predictive value; -=not available. ^a)^Weighted pooled based on the highest-level medical institution. Adapted from the article of Khil et al. (Korean Circ J 2027;57:e40) [2].
